# Alterations of Gene Expression and Glutamate Clearance in Astrocytes Derived from an MeCP2-Null Mouse Model of Rett Syndrome

**DOI:** 10.1371/journal.pone.0035354

**Published:** 2012-04-20

**Authors:** Yasunori Okabe, Tomoyuki Takahashi, Chiaki Mitsumasu, Ken-ichiro Kosai, Eiichiro Tanaka, Toyojiro Matsuishi

**Affiliations:** 1 Division of Gene Therapy and Regenerative Medicine, Cognitive and Molecular Research Institute of Brain Diseases, Kurume University, Kurume, Japan; 2 Department of Physiology, Kurume University of Medicine, Kurume, Japan; 3 Department of Pediatrics, Kurume University of Medicine, Kurume, Japan; 4 Department of Gene Therapy and Regenerative Medicine, Advanced Therapeutics Course, Kagoshima University Graduate School of Medical and Dental Sciences, Kagoshima, Japan; University of Insubria, Italy

## Abstract

Rett syndrome (RTT) is a neurodevelopmetal disorder associated with mutations in the methyl-CpG–binding protein 2 (MeCP2) gene. MeCP2-deficient mice recapitulate the neurological degeneration observed in RTT patients. Recent studies indicated a role of not only neurons but also glial cells in neuronal dysfunction in RTT. We cultured astrocytes from MeCP2-null mouse brain and examined astroglial gene expression, growth rate, cytotoxic effects, and glutamate (Glu) clearance. Semi-quantitative RT-PCR analysis revealed that expression of astroglial marker genes, including GFAP and S100β, was significantly higher in MeCP2-null astrocytes than in control astrocytes. Loss of MeCP2 did not affect astroglial cell morphology, growth, or cytotoxic effects, but did alter Glu clearance in astrocytes. When high extracellular Glu was added to the astrocyte cultures and incubated, a time-dependent decrease of extracellular Glu concentration occurred due to Glu clearance by astrocytes. Although the shapes of the profiles of Glu concentration versus time for each strain of astrocytes were grossly similar, Glu concentration in the medium of MeCP2-null astrocytes were lower than those of control astrocytes at 12 and 18 h. In addition, MeCP2 deficiency impaired downregulation of excitatory amino acid transporter 1 and 2 (EAAT1/2) transcripts, but not induction of glutamine synthetase (GS) transcripts, upon high Glu exposure. In contrast, GS protein was significantly higher in MeCP2-null astrocytes than in control astrocytes. These findings suggest that MeCP2 affects astroglial genes expression in cultured astrocytes, and that abnormal Glu clearance in MeCP2-deficient astrocytes may influence the onset and progression of RTT.

## Introduction

Rett syndrome (RTT) is a neurodevelopmetal disorder that affects one in 15,000 female births, and represents a leading cause of mental retardation and autistic behavior in girls [1], [2]. Mutations in the methyl-CpG–binding protein 2 (MeCP2) gene, located in Xq28, have been identified as the cause for the majority of clinical RTT cases [3]. Knockout mouse models with disrupted MeCP2 function mimic many key clinical features of RTT, including normal early postnatal life followed by developmental regression that results in motor impairment, irregular breathing, and early mortality [4], [5], [6]. MeCP2 dysfunction may thus disrupt the normal developmental or/and physiological program of gene expression, but it remains unclear how this might result in a predominantly neurological phenotype.

In several RTT mouse models, a conditional knockout that is specific to neural stem/progenitor cells or postmitotic neurons results in a phenotype that is similar to the ubiquitous knockout, suggesting that MeCP2 dysfunction in the brain and specifically in neurons underlies RTT [1], [6], [7]. Recent studies have demonstrated that mice born with RTT can be rescued by reactivation of neuronal MeCP2 expression, suggesting that the neuronal damage can be reversed [1], [6]. In addition, several studies using in vitro cell culture systems also indicate that MeCP2 may play a role in processes of neuronal maturation including dendritic growth, synaptogenesis, and electrophysiological responses [1], [7]. These data support the idea that MeCP2 deficiency in neurons is sufficient to cause an RTT-like phenotype. However, emerging evidence now indicates that MeCP2 deficiency in glia may also have a profound impact on brain function [8], [9], [10], [11], [12], [13]. Brain magnetic resonance (MR) studies in MeCP2-deficient mice demonstrated that metabolism in both neurons and glia is affected [8]. Furthermore, in vitro co-culture studies have shown that MeCP2-deficient astroglia non-cell-autonomously affect neuronal dendritic growth [9], [10]. In addition, MeCP2-deficient microglia cause dendritic and synaptic damage mediated by elevated glutamate (Glu) release [11]. Very recent studies have indicated that re-expression of MeCP2 in astrocytes of MeCP2-deficient mice significantly improves locomotion, anxiety levels, breathing patterns, and average lifespan, suggesting that astrocyte dysfunction may be involved in the neuropathology and characteristic phenotypic regression of RTT [13].

Astrocytes regulate the extracellular ion content of the central nervous systems (CNS); they also regulate neuron function, via production of cytokines, and synaptic function, by secreting neurotransmitters at synapses [14], [15]. Moreover, a major function of astrocytes is efficient removal of Glu from the extracellular space, a process that is instrumental in maintaining normal interstitial levels of this neurotransmitter [16]. Glu is a major excitatory amino acid; excess Glu causes the degeneration of neurons and/or seizures observed in various CNS diseases [14], [17]. RTT is also associated with abnormalities in Glu metabolism, but these findings are controversial due to the limitations of the experimental strategies used. Two studies have demonstrated that Glu is elevated in the cerebrospinal fluid (CSF) of RTT patients [18], [19]. MR spectroscopy in RTT patients also revealed elevations of the Glu and Gln peak [20], [21]. On the other hand, an animal MR study reported that the levels of Glu and Gln were decreased in a mouse model of RTT [8]. A more recent study indicated that MeCP2-null mice have reduced levels of Glu, but elevated levels of Gln, relative to their wild-type littermates [22]. Another study reported increased Gln levels and Gln/Glu ratios in Mecp2 mutant mice, but no decreases in Glu levels [23]. Although these in vivo studies have explored the hypothesis that the Glu metabolic systems might be altered in RTT, no solid conclusions have yet been reached [24], [25].

In this study, we investigated the contribution of MeCP2 to the physiological function of astrocytes. Our studies demonstrate that MeCP2 is not essential for the growth and survival of astrocytes, but is involved in astrocytic Glu metabolism via the regulation of astroglial gene expression.

## Results

### Characterization of MeCP2-null astrocytes

**Figure 1:**
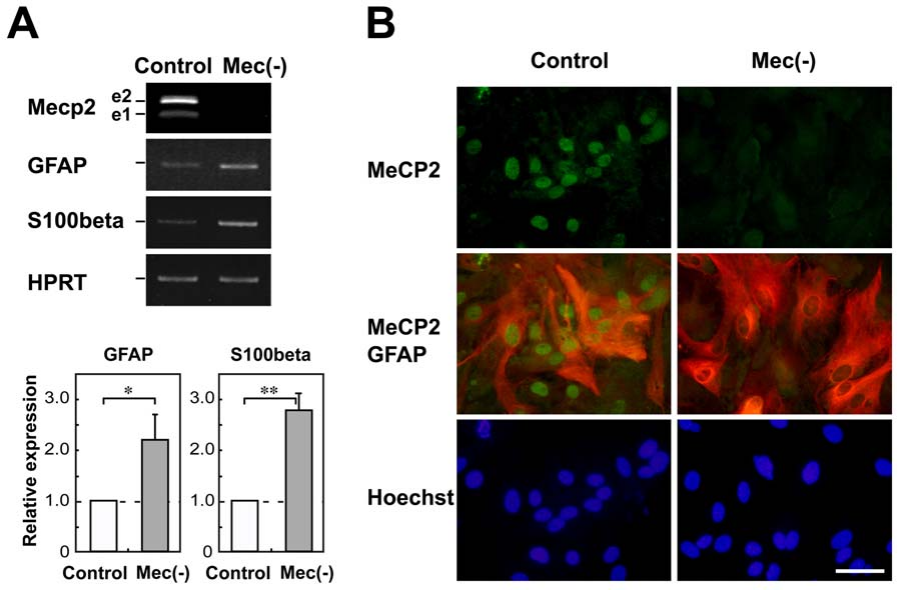
Characterization of assay cultures. **A.** Expression of astroglial genes in primary cultured cortical astrocytes. Semi-quantitative RT-PCR analysis of Mecp2 and astroglial genes was performed in wild-type (white column) and MeCP2-null (gray column) astrocytes. Mecp2 e1 and e2 were detectable in the wild-type astrocytes. The lower graphs show that the GFAP/HPRT or S100β/HPRT expression ratio in each genotype was normalized against the level in control astrocytes. Bars represent the means ± standard errors (SE) of samples from three independent experiments (*p<0.05). The expression of astroglial markers was significantly upregulated by MeCP2 deficiency. **B.** Expression of MeCP2 in the primary cultured cortical astrocytes. The astrocytes were immunostained with MeCP2 (green) and GFAP (red) as glial-specific astrocytic markers. Scale bars indicate 50 µm.

It was recently reported that MeCP2 is normally present not only in neurons but also in glia, including astrocytes, oligodenrocytes, and microglia [9], [10], [11]. To determine the roles of MeCP2 in astrocytes, we cultured cerebral cortex astrocytes from both wild-type (MeCP2^+/y^) and MeCP2-null (MeCP2^−/y^) mouse brains (Fig. 1). MeCP2-null astrocytes exhibited a large, flattened, polygonal shape identical to that of the wild-type astrocytes, suggesting that normal patterns of cellular recognition and contact were present. Semi-quantitative RT-PCR using primer sets that specifically amplify two splice variants, Mecp2 e1 and e2, showed that control astrocytes expressed Mecp2 e1 and e2, whereas neither Mecp2 variant was detectable in MeCP2-null astrocytes (Fig. 1A). We further confirmed expression of MeCP2 by immunocytochemical staining of astrocytes. In control samples, almost all GFAP-positive cells exhibited clear nuclear MeCP2 immunoreactivity in astrocytes, but no immunoreactivity was observed in MeCP2-null astrocytes (Fig. 1B).

MeCP2 has been reported to be involved in regulation of astroglial gene expression [26], [27]. Consistent with this, GFAP levels were significantly higher in MeCP2-null astrocytes (Fig. 1A). Similarly, the expression of S100β, another astrocyte maturation marker, was significantly upregulated by MeCP2 deficiency (fold change of control = 1.0, GFAP: 2.195±0.504, n = 4 each, p<0.05; S100β: 2.779±0.329, n = 4 each, p<0.01). These results show that MeCP2 deficiency upregulates astroglial gene expression in astrocytes.

**Figure 2:**
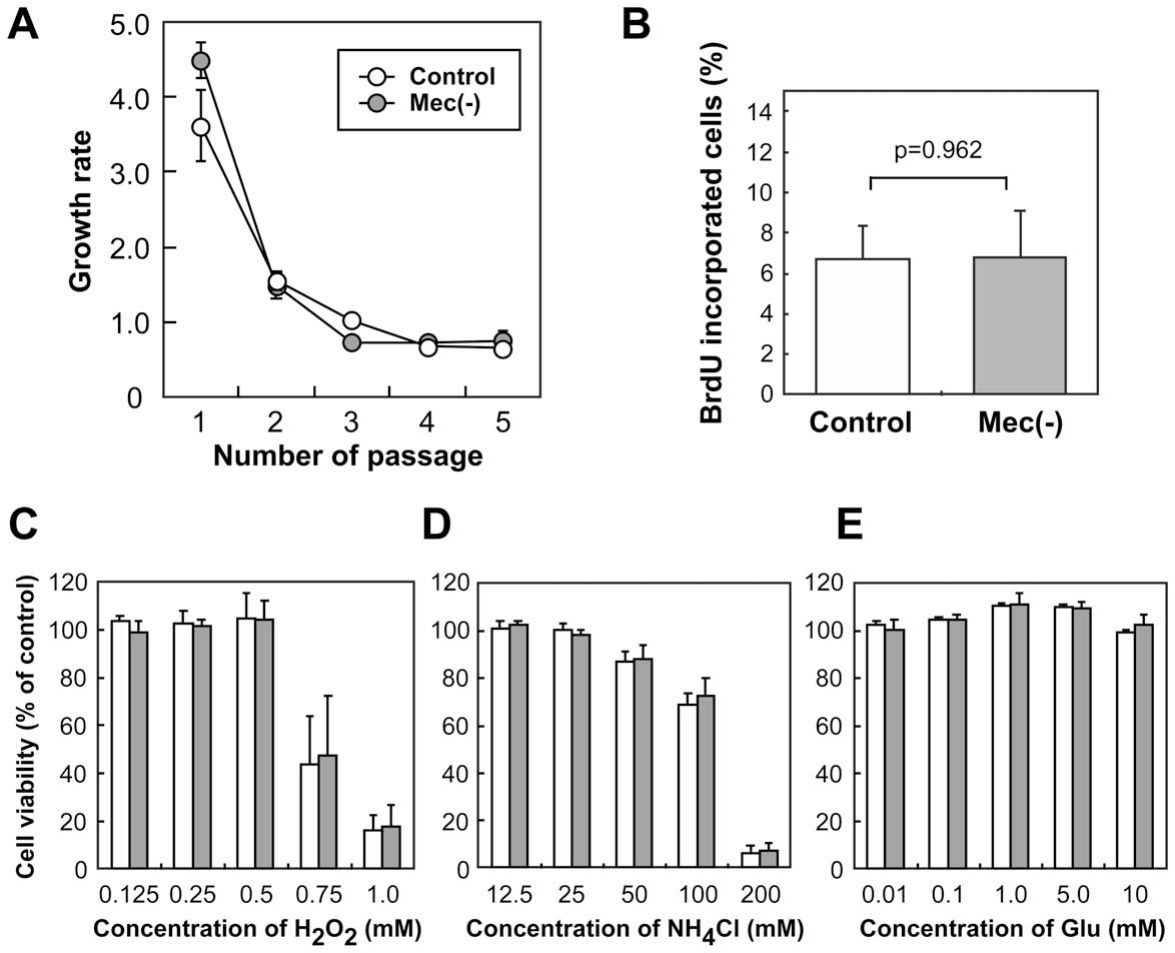
Cell growth and viability. **A.** Comparison of cell growth in wild-type and MeCP2-deficient astrocytes. As passage number increased, cell growth rate decreased dramatically in both strains of astrocytes. There was no significant difference in growth rate between the control and MeCP2-null astrocyte cultures. **B.** Quantification of BrdU-incorporating cells in control and MeCP2-null astrocytes. Astrocytes were cultured for 24 h and incubated with BrdU for 2 h. The graph shows the percentage of BrdU-incorporating cells in the control (white column) and MeCP2-deficient (gray column) astrocytes 2 h after BrdU exposure. The number of BrdU-incorporating cells is expressed as a percentage of the total number of Hoechst-stained cells (Fig. S1). Bars represent the means ± SE of the samples from four independent experiments. The ratio of BrdU-incorporating cells is similar in astrocytes taken from both control and MeCP2-null strains. **C–E.** Comparison of effects of various neurotoxins (**C,** H_2_O_2_; **D,** NH_4_Cl; **E,** Glutamate) on control and MeCP2-null astrocytes. The graph shows the percentage of viability in the control (white column) and MeCP2-deficient (gray column) astrocytes after neurotoxin treatment at the indicated concentrations. Bars represent the means ± SE of samples from three independent experiments. The glial cultures showed no difference in viability between the control and MeCP2-null strains.

To compare the growth of the wild-type and MeCP2-null astrocytes, we counted total cell number at each passage (Fig. 2A). As passage number increased, the cell growth rate decreased dramatically for both types of astrocytes, ultimately culminating in senescence. There was no significant difference in growth rate between the control and MeCP2-null astrocyte cultures. We then measured astrocyte proliferation via BrdU incorporation assay (Fig. 2B and Fig. S1). After 2 h of BrdU treatment, the proportions of BrdU-incorporating cells were similar in the control and MeCP2-null astrocytes (6.635±1.655% in control versus 6.774±2.272% in MeCP2-null astrocytes, n = 4 each, p = 0.962). These results suggest that the absence of MeCP2 did not affect the proliferation of astrocytes in our culture condition.

We also tested the cytotoxic effects of hydrogen peroxide (H_2_O_2_), ammonium chloride (NH_4_Cl), and glutamate (Glu), on astrocytes in our culture (Fig. 2C–E). In cultures derived from both wild-type and MeCP2-null strains, cell viability decreased with increasing concentrations of H_2_O_2_ and NH_4_Cl. In contrast, in our culture conditions, we observed virtually 100% viability of both the control and MeCP2-null astrocytes after 24 h incubation with 10 mM Glu. Glu-induced gliotoxic effects have been previously reported by Chen et al. (2000), and are probably due to distinct differences in culture conditions, specifically the presence of glucose [28]. These results showed that H_2_O_2_ and NH_4_Cl had a similar effect in both strains of astrocytes. There was no significant difference in viability between the control and MeCP2-null astrocyte cultures, indicating that MeCP2 deficiency did not affect astrocyte viability upon treatment with H_2_O_2_ and NH_4_Cl.

### Effects of glutamate on glutamate transporters and glutamine synthetase transcripts in MeCP2-null astrocytes

**Figure 3:**
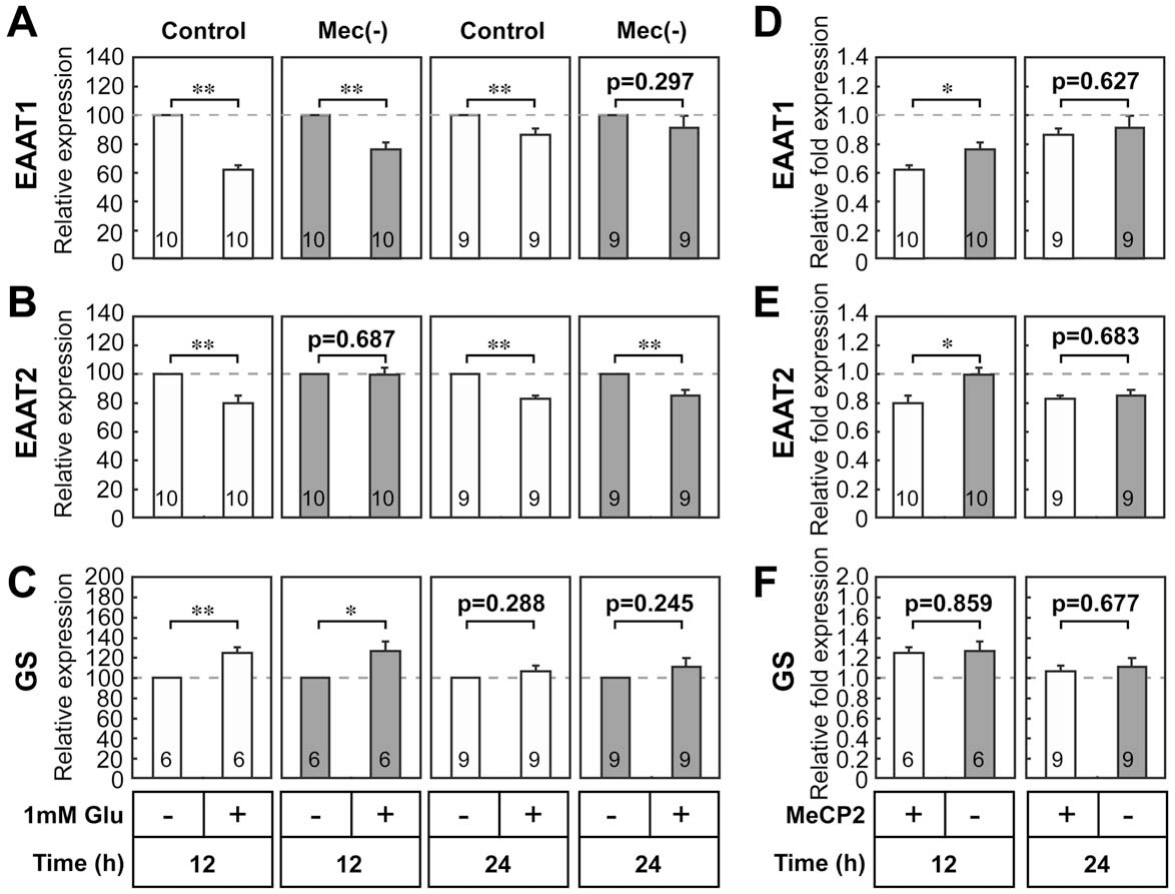
Effect of glutamate on glutamine synthetase and glutamate transporter gene expression in MeCP2-null astrocytes. **A–C.** Effects of Glu on Glu clearance-related genes in wild-type (white column) and MeCP2-null (gray column) astrocytes. Semi-quantitative RT-PCR analysis of Glu clearance-related genes, EAAT1 (**A**), EAAT2 (**B**), and GS (**C**), was performed in the control and MeCP2-null astrocytes 12 or 24 h after treatment with 1.0 mM Glu. The bands corresponding to PCR products were quantified by densitometry, normalized against HPRT levels, and expressed as % of controls (equals 100%). Bars represent the means ± SE of samples from 3–4 independent experiments (*p<0.05, **p<0.01). **D–F.** Comparison of the effects of Glu on EAAT1, EAAT2 or GS expression in the control and MeCP2-null astrocytes. The ratio of EAAT1/HPRT (**D**), EAAT2/HPRT (**E**) or GS/HPRT (**F**) in each treatment group was normalized against that of the non-treated astrocytes from each group. Bars represent the means ± SE of samples from 3–5 independent experiments (*p<0.05). Numbers in each column indicate the total number of samples analyzed.

High extracellular Glu interferes with the expression of the astrocyte transporter subtypes, excitatory amino acid transporter 1(EAAT1)/glutamate/aspartate transporter (GLAST) and EAAT2/glutamate transporter-1 (GLT-1) [16], [29]. To explore the effects of Glu on the expression of Glu transporter genes in cultured astrocytes from wild-type and MeCP2-null mouse brains, we asked whether treatment with 1.0 mM Glu altered expression of EAAT1 and EAAT2 mRNA, using a semi-quantitative RT-PCR assay (Fig. 3). EAAT1 and EAAT2 mRNA were expressed in both wild-type and MeCP2-null astrocytes, and were slightly higher in controls than in MeCP2-null astrocytes. Both EAAT1 and EAAT2 mRNA levels were altered in the control astrocytes after treatment with 1.0 mM Glu. EAAT1 mRNA levels decreased significantly in the wild-type astrocytes, both 12 h and 24 h after treatment with Glu (Fig. 3A). In contrast, EAAT1 decreased significantly in the MeCP2-null astrocytes, at 12 h but not 24 h after treatment. As with EAAT1, EAAT2 mRNA levels also decreased significantly in the control astrocytes, both 12 h and 24 h after treatment (Fig. 3B). However, EAAT2 decreased significantly in MeCP2-null astrocytes, 24 h but not 12 h after treatment. In addition, the effects of Glu on EAAT1 and EAAT2 relative fold expression at 12 h were altered in the MeCP2-null astrocytes (Fig. 3D: EAAT1; 0.618±0.033 in control versus 0.758±0.049 in MeCP2-null astrocytes, n = 10 each, p<0.05; Fig. 3E: EAAT2; 0.794±0.055 in control versus 0.964±0.048 in MeCP2-null astrocytes, n = 8 each, p<0.05). These results suggest that the loss of MeCP2 leads to transcriptional dysregulation of these genes, either directly or indirectly.

One important enzyme that plays a role in the Glu metabolic pathway is glutamine synthetase (GS) [17], [29]. GS is mainly located in astrocytes; cultured astrocytes response to Glu with increased GS expression [17], [29]. Consistent with this, 1.0 mM Glu treatment stimulated GS mRNA expression in both the wild-type and MeCP2-null astrocytes about 1.2-fold after 12 h but not 24 h (Fig. 3C). In addition, MeCP2 deficiency did not modify the effects of Glu on GS mRNA relative fold expression in cultured astrocytes (Fig. 3F, 1.245±0.054 in control versus 1.265±0.093 in MeCP2-null astrocytes, n = 6 each, p = 0.859). These results suggested that MeCP2 did not modify the expression of GS in the cultured astrocytes. Overall, the expression levels of GS mRNA did not differ between both strains of astrocytes following treatment with Glu.

### Comparison of glutamate clearance between wild-type and MeCP2-null astrocytes

**Figure 4:**
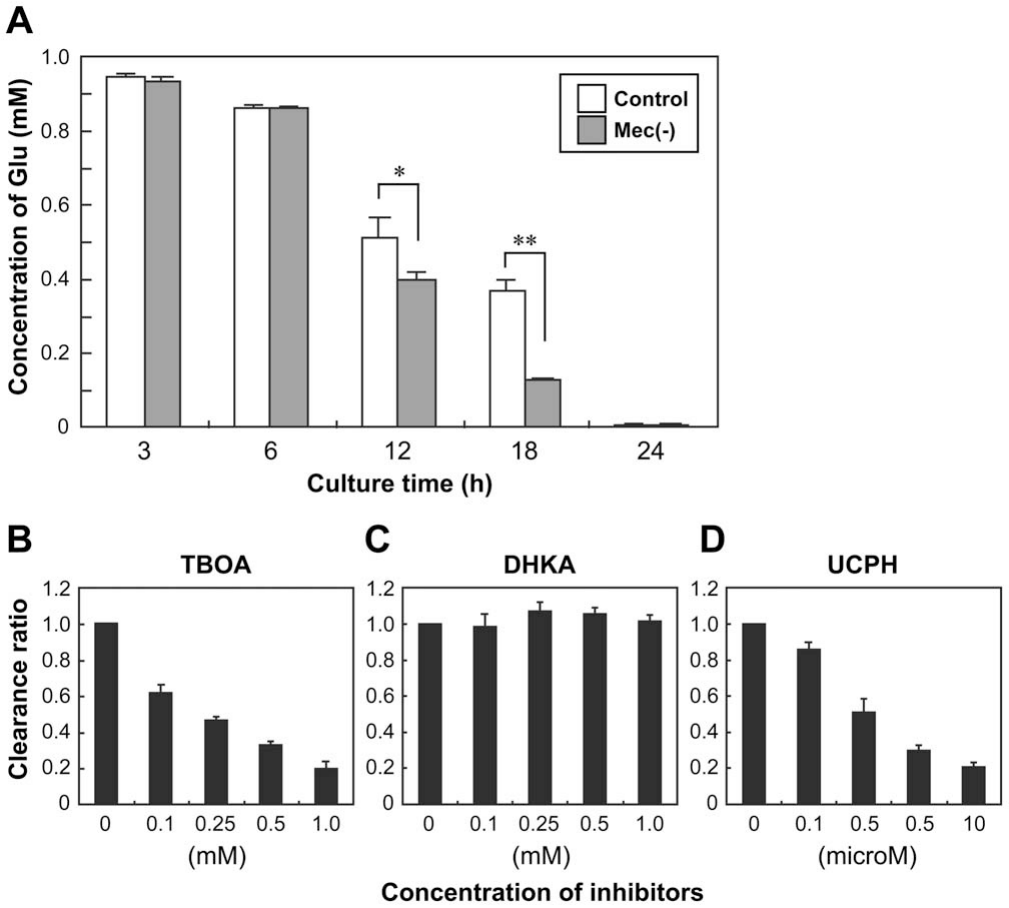
Comparison of glutamate clearance in wild-type and MeCP2-null astrocytes. **A.** Time-dependent reduction of extracelluar Glu concentration in wild-type (white column) and MeCP2-null (gray column) astrocyte cultures. After treatment with 1.0 mM Glu, culture supernatant was collected at the indicated times for the determination of Glu concentration. The graph shows the concentration of Glu in control and MeCP2-null astrocyte culture medium. Bars represent the means ± SE of samples from three independent experiments (*p<0.05). **B–D.** Effects of inhibitors of glutamate transporters (**B,** TBOA; **C,** DHKA; **D,** UCPH) on Glu clearance. Astrocytes were exposed to the indicated concentration of Glu transporter inhibitors, and then 0.1 mM Glu was added; culture supernatant was collected for the determination of Glu concentration at 2 h. The graphs show the clearance ratio upon treatment with each inhibitor. The clearance ratio in the indicated concentration groups was expressed by defining the control level (no inhibitor) as 1.0. Bars represent the means ± SE of samples from three independent experiments.

Because MeCP2 contributed to the transcriptional regulation of Glu metabolism-related genes in our culture systems, we next compared the Glu clearance capability of the wild-type and MeCP2-null astrocytes (Fig. 4A). The cell culture supernatants in both astrocyte cultures were collected at 3–24 h post incubation in culture media containing 1.0 mM Glu. After incubation in culture medium containing Glu, we identified a time-dependent reduction in Glu over 24 h of incubation in both strains of astrocytes. Although the shapes of the profiles of Glu concentration versus time for each strain of astrocytes were grossly similar, Glu concentration in the medium of MeCP2-null astrocytes were lower than those of control astrocytes at 12 and 18 h (12 h: control, 0.513±0.052 mM versus MeCP2-null, 0.395±0.022 mM, p<0.05; 18 h: control, 0.368±0.029 mM versus MeCP2-null, 0.125±0.007 mM, p<0.01, n = 6 each, Fig. 4A). The differences in Glu clearance were not due to changes in cell death of control astrocytes upon application of Glu (Fig. 2E). This indicates that Glu clearance by MeCP2-null astrocytes was more efficient than by control astrocytes.

The Glu transporters EAAT1 and EAAT2 are located primarily on astrocytes and are critical in maintaining extracellular Glu at safe levels [16]. Threo-beta-benzyloxyaspartate (TBOA) is a broad-spectrum glutamate transporter antagonist, affecting EAAT1 and EAAT2 [30]. UCPH-101 (2-amino-4-(4-methoxyphenyl)-7-(naphthalen-1-yl)-5-oxo-5,6,7,8-tetrahydro-4H-chromene-3-carbonitrile) and dihydrokainate (DHKA) are selective inhibitors for EAAT1 and EAAT2, respectively [30], [31]. To investigate the functional Glu transporters in our astrocyte cultures, we analyzed three Glu transporter blockers (TBOA, UCPH-101, or DHKA) for their ability to alter the effects of Glu clearance (Fig. 4B–D). Glu clearance by the wild-type astrocytes was partially blocked by addition of TBOA and UCPH-101, but not DHKA. This suggests that EAAT1, but not EAAT2, plays a major role in Glu clearance under our astroglial culture conditions.

### Effects of glutamate on glutamine synthetase and EAAT1 protein in MeCP2-null astrocytes

**Figure 5:**
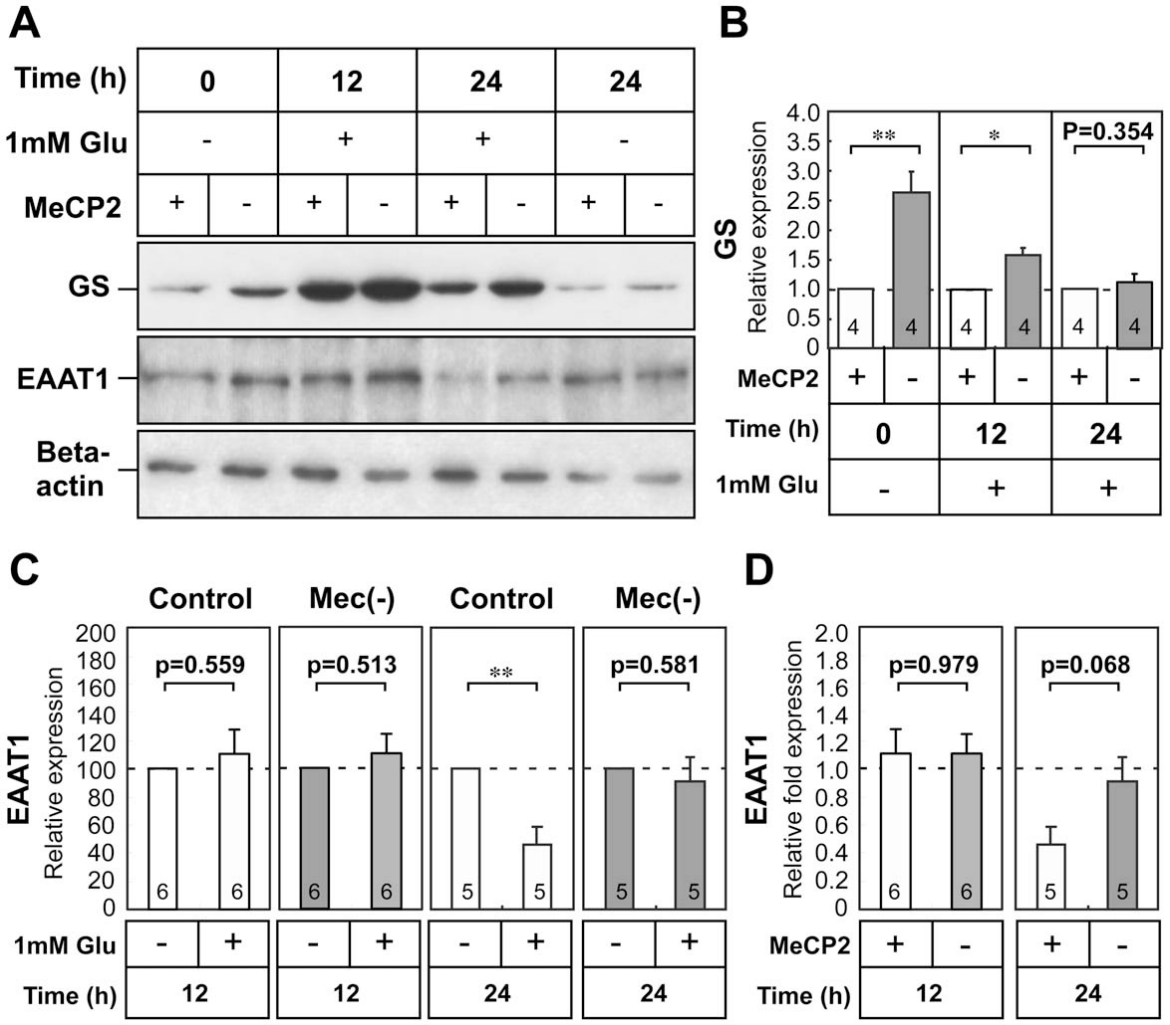
Effect of glutamate on glutamine synthetase and EAAT1 protein expression in MeCP2-null astrocytes. **A.** Time-dependent expression of GS and EAAT1 proteins in wild-type and MeCP2-deficient astrocyte cultures. Astrocytes were treated with 1.0 mM Glu for 24 h, and subsequently analyzed for expression of GS and EAAT1 by Western blot analysis. Beta-actin protein levels were analyzed in the same way, as an internal control. **B.** The immunoreactive GS protein bands were quantified by densitometry, normalized against β-actin levels, and expressed as fold change relative to the controls (equals 1.0). Bars represent the means ± SE of samples from three independent experiments (*p<0.05, **p<0.01). Numbers in each column indicate the total number of samples analyzed. **C.** The immunoreactive EAAT1 protein bands were quantified by densitometry, normalized against β-actin levels, and expressed as % of controls (equals 100%). Bars represent the means ± SE of samples from three independent experiments (**p<0.01). **D.** Comparison of the effects of Glu on EAAT1 expression in wild-type and MeCP2-null astrocytes. The ratio of EAAT1/β-actin in each treatment group was normalized against that of the non-treated astrocytes from each group. Bars represent the means ± SE of samples from three independent experiments. Numbers in each column indicate the total number of samples analyzed.

The initial set of experiments aimed to determine whether Glu modulate the translation of GS and EAAT1 protein (Fig. 5 and Fig. S2). GS protein was expressed in both wild-type and MeCP2-null astrocytes, and was significantly more abundant in MeCP2-null astrocytes (Fig. 5B: fold change of control = 1.0, 2.631±0.368, p<0.01). After 12 h exposure to 0.01–1.0 mM Glu, wild-type astrocytes exhibited a dose-dependent increase in GS protein levels (about 6-fold in 1.0 mM Glu treatment). Similar to its effect on the wild-type astrocytes, in the MeCP2-null astrocytes Glu exposure dose-dependently increased GS protein levels relative to untreated astrocytes (Fig. S2). We then examined the effect of 1.0 mM Glu on levels of GS protein, over a time course (Fig. 5A). GS expression was highest after 12 h exposure to 1.0 mM Glu, decreasing slightly by 24 h in both wild-type and MeCP2-null astrocytes. Densitometric analysis of the bands in three independent experiments demonstrated that GS protein in MeCP2-null astrocyte cultures was higher than in wild-type astrocytes, 12 h but not 24 h after treatment (Fig. 5B: fold change of control = 1.0, at 12 h: 1.421±0.139, p<0.05; at 24 h: 1.131±0.130, p = 0.354, n = 4 each). These results indicated that MeCP2 deficiency caused higher expression of GS protein in cultured astrocytes.

We also asked whether treatment with 1.0 mM Glu altered expression of EAAT1 protein. EAAT1 protein was expressed in both wild-type and MeCP2-null astrocytes, at levels that were similar in controls and MeCP2-null astrocytes. EAAT1 protein levels were altered in the wild-type astrocytes after treatment with 1.0 mM Glu. EAAT1 protein levels decreased significantly in the wild-type astrocytes, 24 h but not 12 h after treatment (Fig. 5C). In contrast, EAAT1 did not decrease in the MeCP2-null astrocytes, either 12 h or 24 h after treatment. In addition, the relative expression levels of EAAT1 24 h after treatment were lower in the wild-type than in the MeCP2-null culture, although the difference was not statistically significant (Fig. 5D: 12 h; 1.102±0.169 in control versus 1.096±0.142 in MeCP2-null astrocytes, n = 6 each, p = 0.979, 24 h; 0.456±0.123 in control versus 0.901±0.172 in MeCP2-null astrocytes, n = 5 each, p = 0.068). These results suggest that MeCP2 deficiency affects the expression of GS and EAAT1 protein, and that accelerated Glu clearance may result from dysregulation of GS and EAAT1 protein in MeCP2-null astrocytes.

## Discussion

Recent studies suggest that glia, as well as neurons, cause neuronal dysfunction in RTT via non-cell-autonomous effects. Here, we have demonstrated that MeCP2 regulates the expression of astroglial marker transcripts, including GFAP and S100β in cultured astrocytes. In addition, MeCP2 is not essential for the cell morphology, growth, or viability; rather, it is involved in Glu clearance through the regulation of Glu transporters and GS in astrocytes. Altered astroglial gene expression and abnormal Glu clearance by MeCP2-null astrocytes may underlie the pathogenesis of RTT.

In this study, MeCP2-null astrocytes exhibited significantly higher transcripts corresponding to astroglial markers, including GFAP and S100β. Consistent with this, transcription of several astrocytic genes, including GFAP, is upregulated in RTT patients [26], [32]. Indeed, MeCP2 binds to a highly methylated region in the GFAP and S100β in neuroepithlial cells [27], [33]; ectopic overexpression of MeCP2 inhibited the differentiation of neuroepithelial cells into GFAP-positive glial cells [34]. Our recent study in RTT-model ES cells also demonstrated that MeCP2 is involved in gliogenesis during neural differentiation via inhibition of GFAP expression [12]. Therefore, MeCP2 may be involved not only in the suppression of astroglial genes in neuroepithelial cells/neurons during neurogenesis, but also in the physiological regulation of astroglial gene expression in astrocytes.

We also demonstrated that MeCP2 is not essential for cell growth or cell viability in in vitro models of astrocyte injury, such as H_2_O_2_ oxidative stress and ammonia neurotoxicity. On the other hand, it has been reported that MeCP2 is involved in regulating astrocyte proliferation, and are probably due to distinct differences in culture conditions, specifically the presence of serum [10]. Consistent with these results, obvious neuronal and glial degeneration had not been observed in RTT [6], [35]. These observations suggest that RTT is not caused by reduced cell numbers, but rather by dysfunction of specific cell types in the brain.

The regulation of Glu levels in the brain is an important component of plasticity at glutamatergic synapses, and of neuronal damage via excessive activation of Glu receptors [15], [16]. Astrocytic uptake of Glu, followed by conversion of Glu to Glutamine (Gln), is the predominant mechanism of inactivation of Glu once it has been released in the synaptic cleft. This uptake involves two transporters, EAAT1/GLAST and EAAT2/GLT-1 [16]. Increases in extracellular Glu, present in many brain injuries, are sufficient to modulate the expression of Glu transporters and GS [16], [29]. Furthermore, application of 0.5–1.0 mM Glu to cultured cortical astrocytes causes a decline in EAAT1/GLAST and EAAT2/GLT-1 expression [29]. Our present studies reveal that 1.0 mM extracellular Glu is sufficient to inhibit astroglial Glu transporter expression and to stimulate GS expression in control astrocytes. However, such regulatory influences on Glu transporters are impaired by MeCP2 deficiency. Therefore, MeCP2 may regulate the expression of Glu transporters under physiological conditions. Currently, little is known about the promoter regions of the main Glu transporters [36], [37]. Promoter analysis in each gene may help to elucidate the complex regulations of astroglial genes by MeCP2.

On the other hand, in our culture conditions, MeCP2 deficiency did not impair the expression of GS transcripts in cultured astrocytes, but did affect the expression of GS protein. A very recent study has shown that defects in the AKT/mTOR pathway are responsible for altered translational control in MeCP2 mutant neuron [38]. These findings suggest that a deficit in protein synthesis and/or turnover in the MeCP2-null astrocytes might influence the final levels of GS protein. Further studies are necessary to investigate whether MeCP2 deficiency impairs the synthesis and turnover of proteins in RTT.

The most important finding in this study was that MeCP2 deficiency in astrocytes accelerates Glu clearance. Consistent with this, RTT is associated with abnormalities in the Glu metabolism [24]. Some studies have demonstrated increases in Glu levels in the cerebrospinal fluid (CSF) of human RTT patients [18], [19]. On the other hand, in animal studies there have been instances of decreased Glu levels and/or Glu/Gln ratios, as determined by in MR spectroscopy [8], [21], [22], [23]. Furthermore, MeCP2-deficient microglia release an abnormally high level of Glu, causing excitotoxicity that may contribute to dendritic and synaptic abnormalities in RTT [11]. These results clearly suggest that MeCP2 has the potential to regulate Glu levels in the brain under certain circumstances. Glu levels are altered in the RTT brain, but the mechanisms responsible for the changes in Glu metabolism are unknown. In light of our findings, we speculate that abnormal expression of Glu transporters and GS resulting from MeCP2 deficiency could lead to abnormal Glu clearance in astrocytes and in turn to altered levels of Glu in RTT brain. Additional studies are needed to determine the mechanisms underlying changes in Glu levels and Glu metabolism, and their role in the RTT brain.

In conclusion, MeCP2 modulates Glu clearance through the regulation of astroglial genes in astrocytes. This study suggests a novel role for MeCP2 in astrocyte function; these findings may be useful in exploration of a new approach for preventing the neurological dysfunctions associated with RTT.

## Materials and Methods

### Cell culture

For each experiment, primary cultures were generated from individual MeCP2-null neonates and their wild-type littermates; tail snips from each neonate were obtained for genotyping, as described below. Enriched cultures of GFAP-expressing astroglial cells, which are virtually free of neurons and microglial cells, were established from the cerebral hemispheres of postnatal day (P) 0 to P1 newborn mice, as previously described [29]. In brief, pieces of dissected tissue were trypsinized (0.05%) for 10 min in Ca_2_^+^- and Mg_2_^+^-free phosphate-buffered saline (PBS) supplemented with 0.02% EDTA. Tissue samples were subsequently dissociated in Hank's balanced salt solution (HBSS) containing 15% fetal calf serum (FCS; F2442, Sigma-Aldrich, Inc., St. Louis, MO, USA) by trituration though 10-ml plastic pipettes. Cells were pelleted at 100×g for 5 min, resuspended in Dulbecco's modified Eagle's medium (D-MEM; Wako Pure Chemical Industries, Ltd., Osaka, Japan) containing 15% FCS, and seeded into 100-mm culture dishes previously coated with poly-D-lysine (0.1 mg/ml; Wako Pure Chemical Industries, Ltd., Osaka, Japan). Upon reaching confluency, cells were trypsinized and replated. Cells were used after the third passage (P3) in all experiments, and were seeded at 3×10^4^ cells/cm^2^ in 6-well plate dishes or 35-mm culture dishes. Cultures were assayed by immunochemical analysis using antibodies against GFAP, MAP2, and CD11b in order to determine the degree of enrichment; the astrocyte cultures were nearly pure without contamination of microglia and neurons (Fig. S3 and Information S1).

### Cell growth and bromo-2′-deoxyuridine (BrdU) uptake assay

To determine growth rate, cells were plated at 2×10^5^ cells/dish in 35-mm dishes. At each passage, three dishes per cell line were harvested by trypsinization, and cell numbers were determined using a hemocytometer. Growth rate was expressed as the number of harvested cells divided by the number of seeded cells.

BrdU incorporation during DNA synthesis was determined using the 5-Bromo-2′-deoxy-uridine Labeling and Detection Kit I (Roche, Indianapolis, IN, USA). Briefly, cells were seeded at 3.0×10^4^ cells per well in 48-well culture plates and incubated in D-MEM containing 10% FCS at 37°C for 24 h. After cells were incubated with 10 µM BrdU for 2 h, they were fixed with 70% ethanol in 50 mM glycine (pH 2.0) for 20 min at −20°C. Cells were incubated with an anti-BrdU monoclonal antibody, followed by a fluorescein-coupled goat anti-mouse Ig and Hoechst33324 (1 µg/ml). To determine the percentages of BrdU-positive cells, fluorescent images were obtained by a Biorevo BZ-9000 fluorescence microscope (KEYENCE Co., Osaka, Japan); images were analyzed using the BZ-II application. BrdU-positive cells and total cells were counted in random 3 fields per well (approximately 1200 cells per well). Results were obtained from four independent experiments.

### Cell Viability Analysis

Cell were seeded at 1×10^4^ cells per well in 96-well plates and incubated in D-MEM containing 15% FCS at 37°C for 24 h. In injury models of drug and oxidative stress, cells were incubated with 0.01–10 mM glutamate for 24 h, 12.5–200 mM NH_4_Cl (Sigma Chemical Co.) for 4 h, or 0.125–1.0 mM H_2_O_2_ (Wako Pure Chemical Industry, Osaka, Japan) for 1 h as previously described [28], [39], [40]. After 24 h of drug treatment, cell viability was determined using the WST-8 assay (NACALAI TESQUE, INC., Kyoto, Japan) [39], [41].

### PCR analysis

MeCP2^−/+^ female mice (B6.129P2(C)-Mecp2^tm1.1Bird^/J strain) were purchased from the Jackson Laboratory (Bar Harbor, ME) and mated with wild-type C57BL/6 male mice. DNA samples were extracted from tail snips from newborn animals; prior to nucleic acid extraction, snips were digested with proteinase K. Genotyping was performed by PCR analysis of genomic DNA according to the protocol provided by the manufacturer (http://jaxmice.jax.org/pub-cgi/protocols/protocols.sh?objtype=protocol&protocol_id=598) [4], [12]. All experiments were performed in accordance with the National Institutes of Health Guidelines for the Care and Use of Laboratory Animals, and were approved by the Animal Research Committee of Kurume University.

Total RNA was extracted from cells using a Sepazol RNA I super kit (Nacalai Tesque, Inc., Kyoto, Japan) [41], [42]. One microgram of total RNA was reverse transcribed, and 1/100 of the cDNA (equivalent to 10 ng of total RNA) was subjected to PCR amplification with Taq DNA polymerase (Promega, Co., Ltd., Madison, WI) using the following conditions: 25–35 cycles of 94°C for 30 s, annealing temperature for 60 s, and 74°C for 60 s. Primer sets and annealing temperatures are shown in Table 1. The most appropriate PCR conditions for semi-quantitative analysis of each gene were carefully determined by several preliminary experiments (Fig. S4). The number of cycles for GFAP, S100β, EAAT1, EAAT2, and GS was 25, 32, 35, 32, and 25, respectively (Table 1). The amplified cDNA was electrophoresed on 2% agarose gels containing ethidium bromide, and quantities were analyzed by densitometry using ImageJ software (the Research Service Branch of the National Institute of Health, Bethesda, MD, USA) [42]. The relative expression of each gene was normalized to the intensity of a housekeeping gene, hypoxantine-phosphoribosyl-transferase (HPRT; 30 cycles). The expression level of each gene is reported as a ratio relative to the level of normalized expression in a control sample.

**Table 1:** PCR primers.

	Sense	Antisense	Ta	cycles
GFAP	5′-ATCCGCTCAGGTCATCTTACCC-3′	5′-TGTCTGCTCAATGTCTTCCCTACC-3′	63	25
S100β	5′-AGAGGACTCCAGCAGCAAAGG-3′	5′-AGAGAGCTCAGCTCCTTCGAG-3′	59	32
EAAT1	5′-GAAGTCTCCCAGACGTTCTAATCC-3′	5′-GCTCTGAAACCGCCACTTACTATC-3′	65	35
EAAT2	5′-ATGCTCATCCTCCCTCTTATCATC-3′	5′-CTTTCTTTGTCACTGTCTGAATCTG-3′	63	32
GS	5′-TGTACCTCCATCCTGTTGCC-3′	5′-GTCCCCGTAATCTTGACTCC-3′	57	25
HPRT	5′-CCTGCTGGATTACATTAAAGCACTG-3′	5′-AAGGGCATATCCAACAACAA-3′	57	30
MeCP2	5′-GGTAAAACCCGTCCGGAAAATG-3′	5′-TTCAGTGGCTTGTCTCTGAG-3′	61	35

GFAP, glial fibrillary acidic protein; EAAT, excitatory amino acid transporter; GS, glutamine synthetase; HPRT, hypoxantine-phosphoribosyl-transferase; MeCP2, methyl-CpG-binding protein 2; Ta, annealing temperature (°C).

### Immunocytochemistry

Cultures were fixed with 4% paraformaldehyde for 10 min and permeabilized with 0.05% Triton-X 100 for 5 min. After blocking of nonspecific binding sites with 10% nonfat dry milk in PBS for 1 h, cultures were immunocytochemically stained using antibodies against MeCP2 (anti-MeCP2 polyclonal antibody, MILLIPORE, Temecula, CA, USA; anti-MeCP2 monoclonal antibody, G-6, Santa Cruz Biotechnology, Inc., Santa Cruz, CA), β-tubulin type III (TuJ, Sigma-Aldrich, Inc., St. Louis, Missouri), or glial fibrillary acidic protein (GFAP) (anti-GFAP polyclonal antibody, G9269; anti-GFAP monoclonal antibody, G3893, Sigma-Aldrich, Inc., St. Louis, Missouri), followed by secondary fluorescent antibodies as described previously [12]. Cultures were additionally stained with Hoechst33342 and examined using an Olympus IX-70 (Olympus Japan Inc. Tokyo, Japan) microscope. Photomicrographs were captured using an Olympus DP70 digital camera.

### Immunoblotting

Cell extracts were prepared from astroglial cultures as described previously [41]. Western blot analysis was performed using anti-glutamine synthetase (G2781; Sigma-Aldrich, Inc., St. Louis, Missouri), anti-excitatory amino acid transporter 1 (EAAT1, GLAST; Santa Cruz Biotechnology, Inc., Santa Cruz, CA), horseradish peroxidase-conjugated anti-rabbit IgG (DakoCytomation, Glostrup, Denmark), and chemiluminescent substrate (Chemi-Lumi One, NACALAI TESQUE, INC., Kyoto, Japan) [12], [41]. Several different exposure times were used for each blot to ensure linearity of band intensities. Immunoreactive bands were quantified using the ImageJ software (Research Service Branch of the National Institute of Health, Bethesda, MD, USA). The relative expression of each protein was normalized to the intensity of β-actin. The expression level of each protein is reported as a ratio relative to the level of normalized expression in a control sample.

### Glutamate Clearance Assay

To measure extracellular glutamate (Glu) concentrations, we used the Glutamate Assay Kit colorimetric assay (Yamasa Corporation, Tokyo, Japan) [43]. Assays were carried out in six independent trials. The clearance ratio of Glu was calculated from the Glu concentration (µM) in the medium sample of the drug-treated astroglial cells (Glu_drug_) and the control non-drug treated (i.e., treated with drug vehicle alone) glial cells (Glu_solv_). This is represented mathematically as follows: Glu clearance ratio = (100−Glu_drug_)/(100−Glu_solv_). Threo-beta-benzyloxyaspartate (TBOA), UCPH-101 (2-amino-4-(4-methoxyphenyl)-7-(naphthalen-1-yl)-5-oxo-5,6,7,8-tetrahydro-4H-chromene-3-carbonitrile), or dihydrokainate (DHKA) (all purchased from Tocris Bioscience Ellisville, MO, USA) were applied to astroglial cells 60 min before Glu.

### Statistical analysis

Quantitative results are expressed as means ± standard errors (SE). Student's t-test was used to compare data, with p<0.05 considered significant.

## Supporting Information

Figure S1 **BrdU-incorporating cells in wild-type and MeCP2-null astrocytes.** The top and bottom pictures show BrdU-incorporating (Green) and Hoechst-stained (Blue) cells, respectively, which were stained with the primary anti-BrdU antibodies, the secondary fluorescein-coupled antibodies, and Hoechst 33324. Negative controls received identical treatments, but were not exposed to BrdU. Representative pictures were used to accurately count the number of BrdU incorporated cells to assess the efficiency of astrocyte cell growth. Scale bar = 200 µm.
(EPS)

Figure S2 **Concentration dependency of GS and EAAT1 expression in wild-type and MeCP2-null astrocytes treated with Glutamate.** The astrocytes of each group were treated with 0.01–1.0 mM Glu for 12 h, and subsequently analyzed for expression of GS and EAAT1 by western blot analysis.
(EPS)

Figure S3 **Purity of astroglial cultures from mouse brain.** The purity of astroglial cultures was assessed by immunocytochemisty (**A**) and immunoblotting (**B**) using antibodies against glial fibrillary acidic protein (GFAP; astrocyte marker; Sigma-Aldrich), CD11b (microglial marker; Santacruz), or microtubule associated protein 2 (MAP2; neuronal marker; Sigma-Aldrich). **A.** Immunocytochemistry indicates that neither CD11b nor MAP2 were expressed in astrocyte cultures. Positive control indicates microglia and mouse ES-derived neural cells that stained with anti-CD11b and MAP2 antibodies, respectively. Scale bar = 100 µm. **B.** Western blot analysis of protein extracts from cultured astrocytes and mouse whole brain. Western blot analysis also confirmed that the cultured astrocytes expressed GFAP, but did not express CD11b and MAP2.
(EPS)

Figure S4 **Optimization of the semi-quantitative RT-PCR assay.** Total RNA extracted from neonatal mouse brain astrocytes was serially diluted (2.5, 5, 10, 20, and 40 ng RNA in lanes 1, 2, 3, 4, and 5, respectively), reverse-transcribed and used as control samples in semi-quantitative RT-PCR for GFAP (**A**), S100β (**B**), HPRT (**C**), EAAT1 (**D**), EAAT2 (**E**), and GS (**F**). PCR was carried out for indicated cycles using each of primer sets shown in Table 1. The amplified cDNA was electrophoresed in a 2% agarose gel containing ethidium bromide. NT, RT-PCR with no template.
(EPS)

Information S1 **Supporting materials and methods.**
(DOC)
