# Supplementary material for: Alterations of Gene Expression and Glutamate Clearance in Astrocytes Derived from an MeCP2-Null Mouse Model of Rett Syndrome
Source: PLoS One. 2012 Apr 20;7(4):e35354. doi: 10.1371/journal.pone.0035354 (PMC3332111; doi:10.1371/journal.pone.0035354)
Supplement: Information S1 — Supporting materials and methods. (DOC) [file pone.0035354.s005.doc]

**Supporting Materials and Methods**

**Microglial culture**

Mixed glial cell culture was prepared following the described method [1] with some modifications. For culture of enriched microglia, cerebral hemispheres were minced, dissociated with trypsin, and then maintained with D-MEM (Wako Pure Chemical Industries, Ltd., Osaka, Japan) supplemented with 10% FCS (Sigma-Aldrich, Inc., St. Louis, MO, USA). After 10 day of culture, microglia were detached from an astrocyte monolayer by gently shaking the culture flask. The floating cells were reseeded in 8-well chamber slides (Nalge Nunc internatinal, Naperville, IL) in the same medium.

**ES cell culture and neural differentiation**

D3-ES cells were maintained and differentiated as previously described [2, 3], with some minor modifications. To initiate embryoid body (EB) formation and differentiation, ES-D3 cells were cultured without leukemia inhibitory factor (LIF, ESGRO; Chemicon, Temecula, CA, USA) in low-attachment petri dishes and incubated for 8 days. Medium was changed every 2 days and 5μM retinoic acid (Sigma-Aldrich, Inc., St. Louis, MO, USA) was added after 4 days. EBs were then dissociated and the ES-derived neural cells plated on Poly-D-lysine- and laminin-coated 8-well chamber slides (Nalge Nunc internatinal) in D-MEM supplemented with N2 (GIBCO/BRL, Grand Island, NY, USA).

**Purity analysis of astroglial cultures**

Protein extracted from astroglial cultures and mouse adult whole brain, and performed Western blot analysis using anti-GFAP polyclonal antibodies, anti-CD11b polyclonal antibodies (Santa Cruz biotechnology Inc, Santa Cruz, CA, USA), and anti-MAP2 monoclonal antibodies (Sigma-Aldrich, Inc., St. Louis, MO, USA), and detected with secondary horseradish peroxidase-conjugated antibodies (DakoCytimatin, Glostrup, Denmark) and chemiluminescent substrate (Chemi-Lumi One, NAKALAI TESQUE, INC., Kyoto, Japan). For immuncytochemistry, cells were stained using antibodies against CD11b or MAP2, together with secondary fluorescent antibodies.

**References**

1. Giulian D, Baker TJ. (1986) Characterization of Ameboid Microglia Isolated from Developing Mammalian Brain. J Neurosci 6: 2163-2178

2. Takahashi T, Kawai T, Ushikoshi H, Nagano S, Oshika H, et al. (2006) Identification and isolation of embryonic stem cell-derived target cells by adenoviral conditional targeting. Mol Ther 14: 673-683.

3. Bibel M, Richter J, Schrenk K, Tucker KL, Staiger V, et al. (2004) Differentiation of embryonic stem cells into a defiend neural lineage. Nat Neurosci 7: 1003-1009
